# Supplementary material for: A rare ORAI1 missense variant associates with risk of vascular diseases in White British adults
Source: PLoS One. 2026 Feb 13;21(2):e0337519. doi: 10.1371/journal.pone.0337519 (PMC12904380; doi:10.1371/journal.pone.0337519)
Supplement: S1 Table — (PDF) [file pone.0337519.s001.pdf]

S1 Table: Description of the cardiovascular disease outcomes and their associations with the ORAI1 nonsynonymous SNP, rs3741596.

Disease trait	UK Biobank phenotype definition	Controls	Cases	P-value	OR
**Peripheral vascular disease**	Hospitalisation for PVD with ICD10 code (I739) as a primary diagnosis	429,768	860	0.012	1.7
**Generalised and unspecified atherosclerosis**	Hospitalised for generalised and unspecified atherosclerosis (ICD10 of I709) as a primary diagnosis	430,595	33	0.001	6.8
**Acute ischaemic heart disease**	Hospitalisation for acute ischaemic heart disease, unspecified, with ICD10 code (I249) as a primary diagnosis	430,192	436	0.0497	1.8

ICD10, International Classification of Diseases 10th Revision; OR, Odds Ratio
