# Supplementary material for: A rare ORAI1 missense variant associates with risk of vascular diseases in White British adults
Source: PLoS One. 2026 Feb 13;21(2):e0337519. doi: 10.1371/journal.pone.0337519 (PMC12904380; doi:10.1371/journal.pone.0337519)
Supplement: S2 Table — (PDF) [file pone.0337519.s002.pdf]

S2 Table: Description of cardiac conduction traits and their association with the ORAI1 nonsynonymous SNP, rs3741596.

Disease trait	UK Biobank phenotype definition	Controls	Cases	P-value	OR
**Atrioventricular and left bundle branch block**	Hospitalisation for atrioventricular and left bundle-branch block (ICD10: I44)	429,395	1,233	0.0002	1.9
**Cardiac arrest**	Hospitalisation for cardiac arrest with ICD10 code (I46) as a primary diagnosis	430,324	304	0.54	0.7
**Paroxysmal tachycardia**	Hospitalisation for paroxysmal tachycardia with ICD10 code (I47) as a primary diagnosis	428,167	2,461	0.19	1.2
**Atrial fibrillation**	Hospitalisation for atrial fibrillation and flutter (ICD10 code: I48)	420,971	9,657	0.85	0.98

ICD10, International Classification of Diseases 10th Revision; OR, Odds Ratio
