# Supplementary material for: A rare ORAI1 missense variant associates with risk of vascular diseases in White British adults
Source: PLoS One. 2026 Feb 13;21(2):e0337519. doi: 10.1371/journal.pone.0337519 (PMC12904380; doi:10.1371/journal.pone.0337519)
Supplement: S3 Table — (PDF) [file pone.0337519.s003.pdf]

**S3 Table: The association of rs3741596, with obesity and insulin dependent diabetes mellitus.**

| Disease trait | UK Biobank phenotype definition | Controls | Cases | P-value | OR |
|---|---|---|---|---|---|
| **Obesity** | Hospitalisation for obesity as a primary diagnosis (ICD10: E66) | 430,011 | 617 | 0.27 | 1.4 |
| **Insulin dependent diabetes mellitus** | Hospitalisation for insulin dependent diabetes mellitus with ICD10 code (E10) as a primary diagnosis | 429,881 | 747 | 0.1 | 0.5 |

ICD10, International Classification of Diseases 10$^{th}$ Revision; OR, Odds Ratio
