# Supplementary material for: A rare ORAI1 missense variant associates with risk of vascular diseases in White British adults
Source: PLoS One. 2026 Feb 13;21(2):e0337519. doi: 10.1371/journal.pone.0337519 (PMC12904380; doi:10.1371/journal.pone.0337519)
Supplement: S4 Table — (PDF) [file pone.0337519.s004.pdf]

S4 Table: ORAI1 variants with MAF less than 0.1% associated with peripheral vascular disease.

SNP ID	REF	ALT	Type	OR	LOG(OR)_SE	L95	U95	P
rs149939600	C	T	intronic	1.68	0.23	1.07	2.64	0.024
rs150469021	C	T	intronic	1.74	0.22	1.13	2.67	0.012
rs57017551	C	T	intronic	1.74	0.22	1.13	2.67	0.012
rs181419718	G	C	intronic	1.74	0.22	1.13	2.67	0.012
rs371012412	T	C	intronic	1.73	0.22	1.12	2.66	0.013
rs76452472	C	T	intronic	1.74	0.22	1.13	2.67	0.012
rs75764501	G	A	intronic	1.74	0.22	1.13	2.67	0.012
rs74936888	T	C	intronic	1.74	0.22	1.13	2.67	0.012
rs77568467	G	A	intronic	1.73	0.22	1.12	2.66	0.013
rs114428734	T	C	intronic	1.74	0.22	1.13	2.67	0.012
rs3741596	A	G	exonic nonsynonymous	1.73	0.22	1.13	2.66	0.012
rs3741597	T	C	exonic synonymous	1.73	0.22	1.12	2.66	0.013
rs3825174	T	C	exonic synonymous	1.74	0.22	1.13	2.67	0.012
rs11548651	T	A	UTR3	1.74	0.22	1.13	2.67	0.012
rs76753792	C	T	UTR3	1.74	0.22	1.13	2.67	0.012
rs74808898	A	G	downstream	1.74	0.22	1.13	2.67	0.012
rs75187483	C	G	downstream	1.74	0.22	1.13	2.67	0.012

REF, reference allele; ALT, alternative allele; OR, Odds Ratio; L95, lower 95% confidence interval; U95, lower 95% confidence interval; UTR3, three prime untranslated region
