# Supplementary material for: A rare ORAI1 missense variant associates with risk of vascular diseases in White British adults
Source: PLoS One. 2026 Feb 13;21(2):e0337519. doi: 10.1371/journal.pone.0337519 (PMC12904380; doi:10.1371/journal.pone.0337519)
Supplement: S5 Table — (PDF) [file pone.0337519.s005.pdf]

S5 Table: ORAI1 variants with MAF less than 0.1% associated with generalised atherosclerosis.

SNP ID	REF	ALT	Type	OR	LOG(OR)_SE	L95	U95	P
rs149939600	C	T	intronic	7.15	0.58	2.29	22.30	0.001
rs6486786	T	C	intronic	1.65	0.25	1.02	2.68	0.042
rs6486787	G	C	intronic	2.06	0.26	1.23	3.46	0.006
rs150469021	C	T	intronic	6.79	0.58	2.17	21.23	0.001
rs6486789	T	C	intronic	1.74	0.25	1.07	2.82	0.025
rs7135617	T	G	intronic	1.66	0.25	1.02	2.70	0.040
rs57017551	C	T	intronic	6.79	0.58	2.17	21.23	0.001
rs7484839	C	T	intronic	1.87	0.29	1.05	3.31	0.032
rs181419718	G	C	intronic	6.79	0.58	2.17	21.23	0.001
rs7968061	T	C	intronic	1.94	0.28	1.12	3.36	0.018
rs3892486	G	A	intronic	2.12	0.28	1.23	3.68	0.007
rs7956644	G	A	intronic	2.04	0.27	1.22	3.44	0.007
rs371012412	T	C	intronic	6.78	0.58	2.17	21.21	0.001
rs6486790	A	G	intronic	2.03	0.27	1.21	3.42	0.008
rs7398511	C	A	intronic	1.88	0.28	1.09	3.22	0.023
rs76452472	C	T	intronic	6.79	0.58	2.17	21.23	0.001
rs10522094	C	CAGGG	intronic	1.76	0.25	1.08	2.87	0.022
rs12300327	A	G	intronic	2.06	0.26	1.23	3.46	0.006
rs75764501	G	A	intronic	6.79	0.58	2.17	21.22	0.001
rs527936854	C	T	intronic	11.71	1.08	1.41	97.25	0.023
rs6486795	T	C	intronic	1.99	0.26	1.19	3.33	0.009
rs74936888	T	C	intronic	6.79	0.58	2.17	21.22	0.001
rs11043296	C	T	intronic	1.87	0.29	1.06	3.31	0.032
rs77568467	G	A	intronic	6.75	0.58	2.16	21.12	0.001
rs11043306	T	C	intronic	1.87	0.29	1.06	3.31	0.032
rs114428734	T	C	intronic	6.79	0.58	2.17	21.23	0.001
rs3741596	A	G	exonic nonsynonymous	6.77	0.58	2.16	21.16	0.001

rs3741597	T	C	exonic synonymous	6.77	0.58	2.17	21.18	0.001
rs3825174	T	C	exonic synonymous	6.79	0.58	2.17	21.23	0.001
rs3825175	T	C	exonic synonymous	1.67	0.25	1.03	2.71	0.039
rs11548651	T	A	UTR3	6.79	0.58	2.17	21.23	0.001
rs76753792	C	T	UTR3	6.79	0.58	2.17	21.23	0.001
rs35558190	A	AT	UTR3	1.86	0.28	1.08	3.21	0.025
rs74808898	A	G	downstream	6.79	0.58	2.17	21.23	0.001
rs75187483	C	G	downstream	6.79	0.58	2.17	21.23	0.001

REF, reference allele; ALT, alternative allele; OR, Odds Ratio; L95, lower 95% confidence interval; U95, lower 95% confidence interval; UTR3, three prime untranslated region
