# Supplementary material for: A rare ORAI1 missense variant associates with risk of vascular diseases in White British adults
Source: PLoS One. 2026 Feb 13;21(2):e0337519. doi: 10.1371/journal.pone.0337519 (PMC12904380; doi:10.1371/journal.pone.0337519)
Supplement: S6 Table — (PDF) [file pone.0337519.s006.pdf]

S6 Table: ORAI1 variants with MAF less than 0.1% associated with acute ischaemic heart disease.

SNP ID	REF	ALT	Type	OR	LOG(OR)_SE	L95	U95	P
rs149939600	C	T	intronic	1.93	0.30	1.06	3.50	0.031
rs150469021	C	T	intronic	1.82	0.30	1.00	3.29	0.049
rs573572950	G	A	intronic	3.68	0.38	1.76	7.70	0.001
rs191884363	G	A	intronic	3.35	0.57	1.09	10.26	0.034
rs140061932	G	C	intronic	3.43	0.38	1.63	7.22	0.001
rs577349278	C	T	intronic	4.02	0.59	1.26	12.77	0.018
rs57017551	C	T	intronic	1.82	0.30	1.00	3.29	0.049
rs181419718	G	C	intronic	1.82	0.30	1.00	3.29	0.049
rs76452472	C	T	intronic	1.82	0.30	1.00	3.29	0.048
rs372181267	C	T	intronic	3.43	0.39	1.61	7.31	0.001
rs75764501	G	A	intronic	1.82	0.30	1.00	3.29	0.048
rs74936888	T	C	intronic	1.82	0.30	1.00	3.29	0.048
rs114428734	T	C	intronic	1.82	0.30	1.00	3.29	0.048
rs3741596	A	G	exonic nonsynonymous	1.81	0.30	1.00	3.28	0.0497
rs3741597	T	C	exonic synonymous	1.81	0.30	1.00	3.28	0.0497
rs3825174	T	C	exonic synonymous	1.82	0.30	1.00	3.29	0.048
rs11548651	T	A	UTR3	1.82	0.30	1.00	3.29	0.048
rs76753792	C	T	UTR3	1.82	0.30	1.00	3.29	0.048
rs74808898	A	G	downstream	1.82	0.30	1.00	3.29	0.048
rs75187483	C	G	downstream	1.82	0.30	1.00	3.29	0.048

REF, reference allele; ALT, alternative allele; OR, Odds Ratio; L95, lower 95% confidence interval; U95, lower 95% confidence interval; UTR3, three prime untranslated region
