# Supplementary material for: A rare ORAI1 missense variant associates with risk of vascular diseases in White British adults
Source: PLoS One. 2026 Feb 13;21(2):e0337519. doi: 10.1371/journal.pone.0337519 (PMC12904380; doi:10.1371/journal.pone.0337519)
Supplement: S7 Table — (PDF) [file pone.0337519.s007.pdf]

S7 Table: ORAI1 variants with MAF less than 0.1% associated with atrioventricular and left bundle branch block.

SNP ID	REF	ALT	Type	OR	LOG(OR)_SE	L95	U95	P
rs149939600	C	T	intronic	1.83	0.19	1.28	2.63	0.0011
rs150469021	C	T	intronic	1.91	0.18	1.35	2.69	0.0002
rs57017551	C	T	intronic	1.91	0.18	1.35	2.69	0.0002
rs181419718	G	C	intronic	1.91	0.18	1.35	2.69	0.0002
rs371012412	T	C	intronic	1.96	0.17	1.40	2.75	0.0001
rs76452472	C	T	intronic	1.91	0.18	1.35	2.69	0.0002
rs75764501	G	A	intronic	1.91	0.18	1.35	2.69	0.0002
rs74936888	T	C	intronic	1.91	0.18	1.35	2.69	0.0002
rs77568467	G	A	intronic	1.90	0.18	1.35	2.68	0.0003
rs114428734	T	C	intronic	1.91	0.18	1.35	2.69	0.0002
rs3741596	A	G	exonic nonsynonymous	1.90	0.18	1.35	2.69	0.0002
rs3741597	T	C	exonic synonymous	1.90	0.18	1.35	2.68	0.0003
rs3825174	T	C	exonic synonymous	1.91	0.18	1.35	2.69	0.0002
rs11548651	T	A	UTR3	1.91	0.18	1.35	2.69	0.0002
rs76753792	C	T	UTR3	1.91	0.18	1.35	2.69	0.0002
rs74808898	A	G	downstream	1.91	0.18	1.35	2.69	0.0002
rs75187483	C	G	downstream	1.91	0.18	1.35	2.69	0.0002

REF, reference allele; ALT, alternative allele; OR, Odds Ratio; L95, lower 95% confidence interval; U95, lower 95% confidence interval; UTR3, three prime untranslated region
