# Supplementary material for: A rare ORAI1 missense variant associates with risk of vascular diseases in White British adults
Source: PLoS One. 2026 Feb 13;21(2):e0337519. doi: 10.1371/journal.pone.0337519 (PMC12904380; doi:10.1371/journal.pone.0337519)
Supplement: S8 Table — (PDF) [file pone.0337519.s008.pdf]

S8 Table: Association results of rs3741596 with circulating lipids traits in UK Biobank.

Trait	Cases	BETA	SE	L95	U95	P
Apolipoprotein A	103731	0.0032	0.0058	-0.0083	0.0146	0.59
Apolipoprotein B	103731	-0.0031	0.0052	-0.0133	0.0071	0.56
Average Diameter for HDL Particles	103731	0.0084	0.0049	-0.0012	0.018	0.09
Average Diameter for LDL Particles	103731	0.0043	0.0022	-0.0001	0.0087	0.06
Average Diameter for VLDL Particles	103731	-0.0591	0.031	-0.1198	0.0016	0.06
Cholesterol in Chylomicrons and Extremely Large VLDL	103731	-0.0028	0.0011	-0.005	-0.0007	0.01
Cholesterol in IDL	103731	0.003	0.0054	-0.0076	0.0136	0.58
Cholesterol in Large HDL	103731	0.0061	0.004	-0.0017	0.0138	0.13
Cholesterol in Large LDL	103731	0.0007	0.0073	-0.0136	0.0149	0.93
Cholesterol in Large VLDL	103731	-0.0024	0.0012	-0.0048	-0.00001	0.05
Cholesterol in Medium HDL	103731	0.0029	0.003	-0.0029	0.0087	0.33
Cholesterol in Medium LDL	103731	-0.0019	0.003	-0.0079	0.0041	0.54
Cholesterol in Medium VLDL	103731	-0.0004	0.0017	-0.0038	0.0029	0.80
Cholesterol in Small HDL	103731	-0.0002	0.0016	-0.0033	0.0029	0.89
Cholesterol in Small LDL	103731	-0.0007	0.0011	-0.003	0.0015	0.52
Cholesterol in Small VLDL	103731	-0.0015	0.0013	-0.0041	0.0012	0.27
Cholesterol in Very Large HDL	103731	0.0012	0.0008	-0.0005	0.0028	0.16
Cholesterol in Very Large VLDL	103731	-0.0015	0.0007	-0.003	-0.0001	0.04
Cholesterol in Very Small VLDL	103731	-0.0002	0.0013	-0.0026	0.0023	0.89
Cholesteryl Esters in Chylomicrons and Extremely Large VLDL	103731	-0.0015	0.0006	-0.0026	-0.0003	0.02
Cholesteryl Esters in HDL	103731	0.0083	0.0060	-0.0036	0.0201	0.17
Cholesteryl Esters in IDL	103731	0.0021	0.004	-0.0058	0.01	0.60
Cholesteryl Esters in Large HDL	103731	0.0048	0.0031	-0.0012	0.0109	0.12
Cholesteryl Esters in Large LDL	103731	-0.0003	0.0054	-0.0108	0.0102	0.96
Cholesteryl Esters in Large VLDL	103731	-0.001	0.0006	-0.0022	0.0002	0.09
Cholesteryl Esters in LDL	103731	-0.0032	0.0083	-0.0195	0.0131	0.70
Cholesteryl Esters in Medium HDL	103731	0.0024	0.0024	-0.0022	0.0071	0.30
Cholesteryl Esters in Medium LDL	103731	-0.002	0.0023	-0.0065	0.0024	0.37
Cholesteryl Esters in Medium VLDL	103731	0.0002	0.001	-0.0017	0.0021	0.84
Cholesteryl Esters in Small HDL	103731	0.00004	0.0012	-0.0024	0.0024	0.98
Cholesteryl Esters in Small LDL	103731	-0.0009	0.0009	-0.0026	0.0008	0.31
Cholesteryl Esters in Small VLDL	103731	-0.0011	0.0009	-0.0028	0.0006	0.20
Cholesteryl Esters in Very Large HDL	103731	0.0010	0.0007	-0.0003	0.0023	0.15
Cholesteryl Esters in Very Large VLDL	103731	-0.0007	0.0004	-0.0014	0.0001	0.08
Cholesteryl Esters in Very Small VLDL	103731	0.0001	0.0009	-0.0017	0.0018	0.93
Cholesteryl Esters in VLDL	103731	-0.004	0.0037	-0.0113	0.0034	0.29

Clinical LDL Cholesterol	103731	0.0015	0.0189	-0.0356	0.0386	0.94
Concentration of Chylomicrons and Extremely Large VLDL Particles	103731	-0.0000001	0.00000004	-0.0000002	-0.00000003	0.01
Concentration of HDL Particles	103731	0.00003	0.0001	-0.0001	0.0001	0.65
Concentration of IDL Particles	103731	-0.0000002	0.000002	-0.000004	0.000004	0.93
Concentration of Large HDL Particles	103731	0.00002	0.00002	-0.00001	0.0001	0.18
Concentration of Large LDL Particles	103731	-0.0000011	0.000005	-0.00001	0.00001	0.81
Concentration of Large VLDL Particles	103731	-0.0000003	0.0000001	-0.0000006	-0.00000004	0.02
Concentration of LDL Particles	103731	-0.000004	0.000007	-0.00002	0.00001	0.60
Concentration of Medium HDL Particles	103731	0.00001	0.00002	-0.0000302	0.0001	0.54
Concentration of Medium LDL Particles	103731	-0.000002	0.0000020	-0.0000056	0.000002	0.39
Concentration of Medium VLDL Particles	103731	-0.0000004	0.0000003	-0.000001	0.0000002	0.21
Concentration of Small HDL Particles	103731	-0.00001	0.00003	-0.0001	0.0001	0.69
Concentration of Small LDL Particles	103731	-0.000001	0.000001	-0.000003	0.000001	0.26
Concentration of Small VLDL Particles	103731	-0.000001	0.0000003	-0.000001	0.00000004	0.07
Concentration of Very Large HDL Particles	103731	0.000002	0.000002	-0.000002	0.000007	0.33
Concentration of Very Large VLDL Particles	103731	-0.0000001	0.0000001	-0.0000003	-0.00000003	0.02
Concentration of Very Small VLDL Particles	103731	-0.0000004	0.0000004	-0.000001	0.0000003	0.32
Concentration of VLDL Particles	103731	-0.000002	0.000001	-0.000004	0.0000003	0.10
Degree of Unsaturation	103657	0.0048	0.002	0.0009	0.0087	0.02
Free Cholesterol in Chylomicrons and Extremely Large VLDL	103731	-0.0014	0.0005	-0.0023	-0.0004	0.01
Free Cholesterol in HDL	103731	0.0016	0.0017	-0.0017	0.005	0.34
Free Cholesterol in IDL	103731	0.0009	0.0014	-0.0019	0.0037	0.53
Free Cholesterol in Large HDL	103731	0.0012	0.0009	-0.0005	0.0030	0.16
Free Cholesterol in Large LDL	103731	0.0009	0.002	-0.0029	0.0048	0.63
Free Cholesterol in Large VLDL	103731	-0.0014	0.0006	-0.0026	-0.0001	0.03
Free Cholesterol in LDL	103731	0.0012	0.0031	-0.0049	0.0073	0.70
Free Cholesterol in Medium HDL	103731	0.0004	0.0006	-0.0007	0.0016	0.45
Free Cholesterol in Medium LDL	103731	0.0001	0.0008	-0.0015	0.0018	0.88
Free Cholesterol in Medium VLDL	103731	-0.0006	0.0008	-0.0021	0.0009	0.40
Free Cholesterol in Small HDL	103731	-0.0003	0.0004	-0.0011	0.0006	0.53
Free Cholesterol in Small LDL	103731	0.0001	0.0003	-0.0005	0.0008	0.66
Free Cholesterol in Small VLDL	103731	-0.0004	0.0005	-0.0013	0.0006	0.45
Free Cholesterol in Very Large HDL	103731	0.0002	0.0002	-0.0001	0.0006	0.25
Free Cholesterol in Very Large VLDL	103731	-0.0009	0.0004	-0.0016	-0.0001	0.02
Free Cholesterol in Very Small VLDL	103731	-0.0002	0.0004	-0.001	0.0005	0.53
Free Cholesterol in VLDL	103731	-0.0049	0.0028	-0.0103	0.0006	0.08
HDL Cholesterol	103731	0.0099	0.0077	-0.0052	0.025	0.20

LDL Cholesterol	103731	-0.002	0.0113	-0.0241	0.0202	0.86
Lipoprotein A	326787	-0.0166	0.7372	-1.4614	1.4282	0.98
Monounsaturated Fatty Acids	103657	-0.0444	0.0214	-0.0863	-0.0026	0.04
Phospho-glycerides	103657	-0.0038	0.0098	-0.0231	0.0155	0.70
Phospholipids in Chylomicrons and Extremely Large VLDL	103731	-0.0023	0.0008	-0.0039	-0.0007	0.01
Phospholipids in HDL	103731	0.0049	0.0076	-0.0099	0.0198	0.52
Phospholipids in IDL	103731	0.0004	0.0017	-0.0029	0.0037	0.81
Phospholipids in Large HDL	103731	0.0048	0.0036	-0.0022	0.0118	0.18
Phospholipids in Large LDL	103731	-0.00001	0.0021	-0.0042	0.0042	1.00
Phospholipids in Large VLDL	103731	-0.0023	0.001	-0.0042	-0.0003	0.02
Phospholipids in LDL	103731	-0.0009	0.0036	-0.008	0.0062	0.80
Phospholipids in Medium HDL	103731	0.0006	0.0025	-0.0042	0.0054	0.81
Phospholipids in Medium LDL	103731	-0.0007	0.0011	-0.0028	0.0014	0.52
Phospholipids in Medium VLDL	103731	-0.0014	0.0012	-0.0039	0.001	0.25
Phospholipids in Small HDL	103731	-0.0018	0.0024	-0.0065	0.0029	0.44
Phospholipids in Small LDL	103731	-0.0002	0.0005	-0.0011	0.0007	0.68
Phospholipids in Small VLDL	103731	-0.0009	0.0008	-0.0025	0.0006	0.22
Phospholipids in Very Large HDL	103731	0.0014	0.0011	-0.0007	0.0035	0.20
Phospholipids in Very Large VLDL	103731	-0.0016	0.0007	-0.0029	-0.0003	0.02
Phospholipids in Very Small VLDL	103731	-0.0007	0.0007	-0.002	0.0006	0.30
Phospholipids in VLDL	103731	-0.0092	0.0047	-0.0184	-0.0001	0.05
Polyunsaturated Fatty Acids	103657	-0.0146	0.0204	-0.0546	0.0253	0.47
Remnant Cholesterol Non-HDL Non-LDL Cholesterol	103731	-0.0058	0.0108	-0.027	0.0154	0.59
Sphingomyelins	103657	0.0013	0.0018	-0.0021	0.0048	0.44
Total Cholesterol	103731	0.0021	0.0238	-0.0444	0.0487	0.93
Total Cholesterol Minus HDLC	103731	-0.0078	0.0217	-0.0504	0.0349	0.72
Total Concentration of Lipoprotein Particles	103731	0.00002	0.0001	-0.0001	0.0001	0.74
Total Esterified Cholesterol	103657	0.0034	0.017	-0.03	0.0368	0.84
Total Fatty Acids	103657	-0.0981	0.0621	-0.2197	0.0235	0.11
Total Free Cholesterol	103731	-0.0015	0.0069	-0.0149	0.012	0.83
Total Lipids in Chylomicrons and Extremely Large VLDL	103731	-0.0133	0.0053	-0.0236	-0.0029	0.01
Total Lipids in HDL	103731	0.0122	0.0152	-0.0176	0.042	0.42
Total Lipids in IDL	103731	0.002	0.0073	-0.0123	0.0163	0.79
Total Lipids in Large HDL	103731	0.0105	0.0076	-0.0044	0.0254	0.17
Total Lipids in Large LDL	103731	-0.0008	0.0097	-0.0198	0.0183	0.94
Total Lipids in Large VLDL	103731	-0.0095	0.0045	-0.0183	-0.0008	0.03
Total Lipids in LDL	103731	-0.0053	0.0155	-0.0357	0.025	0.73
Total Lipids in Lipoprotein Particles	103731	-0.0379	0.0423	-0.1209	0.045	0.37
Total Lipids in Medium HDL	103731	0.0025	0.0055	-0.0082	0.0132	0.65
Total Lipids in Medium LDL	103731	-0.0032	0.0043	-0.0116	0.0051	0.45

Total Lipids in Medium VLDL	103731	-0.0078	0.0053	-0.0182	0.0026	0.14
Total Lipids in Small HDL	103731	-0.0032	0.0041	-0.0112	0.0048	0.44
Total Lipids in Small LDL	103731	-0.0013	0.0017	-0.0046	0.002	0.44
Total Lipids in Small VLDL	103731	-0.0059	0.0034	-0.0125	0.0007	0.08
Total Lipids in Very Large HDL	103731	0.0024	0.0019	-0.0014	0.0062	0.21
Total Lipids in Very Large VLDL	103731	-0.0081	0.0034	-0.0148	-0.0014	0.02
Total Lipids in Very Small VLDL	103731	-0.0021	0.0023	-0.0066	0.0023	0.35
Total Lipids in VLDL	103731	-0.0467	0.0222	-0.0902	-0.0033	0.03
Total Phospholipids in Lipoprotein Particles	103731	-0.0048	0.0117	-0.0278	0.0182	0.68
Total Triglycerides	103731	-0.0352	0.0147	-0.0641	-0.0064	0.02
Triglycerides	410234	-0.0207	0.0133	-0.0468	0.0054	0.12
Triglycerides in Chylomicrons and Extremely Large VLDL	103731	-0.0082	0.0034	-0.0149	-0.0015	0.02
Triglycerides in HDL	103731	-0.0027	0.0012	-0.0051	-0.0003	0.03
Triglycerides in IDL	103731	-0.0014	0.0007	-0.0027	-0.0001	0.03
Triglycerides in Large HDL	103731	-0.0004	0.0003	-0.001	0.0002	0.18
Triglycerides in Large LDL	103731	-0.0014	0.0007	-0.0027	-0.0001	0.03
Triglycerides in Large VLDL	103731	-0.0049	0.0023	-0.0094	-0.0003	0.04
Triglycerides in LDL	103731	-0.0025	0.0011	-0.0045	-0.0004	0.02
Triglycerides in Medium HDL	103731	-0.0010	0.0005	-0.002	-0.00004	0.04
Triglycerides in Medium LDL	103731	-0.0007	0.0003	-0.0012	-0.0001	0.01
Triglycerides in Medium VLDL	103731	-0.0059	0.0029	-0.0117	-0.0002	0.04
Triglycerides in Small HDL	17267	-0.0026	0.0011	-0.0048	-0.0004	0.02
Triglycerides in Small LDL	103731	-0.0004	0.0001	-0.0007	-0.0001	0.01
Triglycerides in Small VLDL	103731	-0.0035	0.0015	-0.0065	-0.0005	0.02
Triglycerides in Very Large HDL	103731	-0.0001	0.0001	-0.0003	-0.000002	0.05
Triglycerides in Very Large VLDL	103731	-0.0050	0.0021	-0.0091	-0.0009	0.02
Triglycerides in Very Small VLDL	103731	-0.0013	0.0005	-0.0023	-0.0002	0.02
Triglycerides in VLDL	103731	-0.0287	0.0123	-0.0528	-0.0046	0.02
VLDL Cholesterol	103731	-0.0088	0.0064	-0.0214	0.0038	0.17

Cases, number of cases; BETA, Regression coefficient; SE, Standard error; L95, lower 95% confidence interval; U95, lower 95% confidence interval
