# Supplementary material for: A rare ORAI1 missense variant associates with risk of vascular diseases in White British adults
Source: PLoS One. 2026 Feb 13;21(2):e0337519. doi: 10.1371/journal.pone.0337519 (PMC12904380; doi:10.1371/journal.pone.0337519)
Supplement: S9 Table — (PDF) [file pone.0337519.s009.pdf]

S9 Table: The associations between the ORAI1 nonsynonymous SNP, rs3741596, and blood cell traits.

Trait	Cases	BETA	SE	L95	U95	P
White blood cell (leukocyte) count	417729	0.0006	0.0270	-0.0525	0.0536	0.98
Lymphocyte count	416995	-0.0272	0.0152	-0.0570	0.0027	0.07
Lymphocyte percentage in leukocytes	417000	-0.2249	0.0959	-0.4130	-0.0369	0.02
Monocyte count	416995	0.0010	0.0028	-0.0046	0.0065	0.73
Monocyte percentage in leukocytes	417000	0.0227	0.0347	-0.0452	0.0907	0.51
Neutrophil count	416995	0.0257	0.0186	-0.0107	0.0621	0.17
Neutrophil percentage in leukocytes	417000	0.1850	0.1104	-0.0315	0.4014	0.09
Eosinophil count	416995	0.0020	0.0018	-0.0015	0.0055	0.26
Eosinophil percentage	417000	0.0251	0.0242	-0.0224	0.0725	0.30
Basophil count	416995	-0.0003	0.0007	-0.0016	0.0010	0.65
Basophil percentage in leukocytes	417000	-0.0075	0.0079	-0.0231	0.0080	0.34
Platelet count	417730	1.8339	0.7648	0.3350	3.3328	0.02
Mean platelet (thrombocyte) volume	417725	-0.0376	0.0142	-0.0654	-0.0099	0.01
Platelet distribution width	417725	-0.0164	0.0067	-0.0296	-0.0032	0.02
Platelet crit	417726	0.0007	0.0006	-0.0005	0.0019	0.23
Red blood cell (erythrocyte) count	417733	0.0060	0.0046	-0.0030	0.0151	0.19
Mean corpuscular volume	417731	-0.0554	0.0574	-0.1678	0.0570	0.33
Haematocrit percentage	417733	0.0297	0.0381	-0.0450	0.1043	0.44
Mean corpuscular haemoglobin	417730	-0.0322	0.0238	-0.0788	0.0143	0.18
Mean corpuscular haemoglobin concentration	417726	-0.0141	0.0138	-0.0412	0.0130	0.31
Haemoglobin concentration	417733	0.0043	0.0129	-0.0211	0.0296	0.74
Red blood cell (erythrocyte) distribution width	417731	0.0337	0.0125	0.0091	0.0582	0.01
Reticulocyte count	411012	-0.0006	0.0005	-0.0016	0.0004	0.22
Reticulocyte percentage	411012	-0.0165	0.0118	-0.0397	0.0066	0.16
Mean reticulocyte volume	411013	-0.0037	0.1025	-0.2047	0.1973	0.97
Immature reticulocyte fraction	411013	0.0004	0.0008	-0.0012	0.0020	0.63
High light scatter reticulocyte count	411012	-0.0001	0.0001	-0.0004	0.0002	0.45
High light scatter reticulocyte percentage	411013	-0.0040	0.0043	-0.0123	0.0044	0.35
Nucleated red blood cell count	416985	-0.0003	0.0004	-0.0011	0.0006	0.55
Nucleated red blood cell percentage	416981	-0.0011	0.0053	-0.0115	0.0093	0.83

REF, reference allele; ALT, alternative allele; Cases, number of cases; BETA, Regression coefficient; SE, Standard error; L95, lower 95% confidence interval; U95, lower 95% confidence interval
